# Legal Medicine in England and in Chicago

**Published:** 1859-11

**Authors:** 


### EDITORIAL.

### LEGAL MEDICINE IN ENGLAND AND IN CHICAGO.

Thomas Smethurst was recently tried and sentenced to be hanged for the murder of Miss Bankes. This sentence has been commuted, and he is to be imprisoned for life.

We learn from the *Lancet* that the moral evidence of his guilt was thought uncertain, and that the testimony on which the charge was founded was that of Dr. Alfred Swayne Taylor, author of a standard work on medical jurisprudence, who, on the *inquest*, testified that he found arsenic in the contents of one of the bottles found in the possession of Smethurst. It was this testimony which virtually condemned Smethurst before the trial.

"On application to Brande, this veteran chemist suggested that possibly the copper wire guage used by Dr. Taylor might have contained arsenic." "Such was found to be the fact when the copper used for testing was in turn tested." Such a mistake is naturally calculated to cast doubt upon the value of

Dr. Taylor's authority as an analytical chemist. There are, however, other facts calculated also to throw discredit upon his correctness.

He says, in the late edition of his work, p. 694, "A man may die from strychnia and no trace of it be found in the body." Mr. Taylor makes this statement apparently as a defence of his position in the case of Palmer, in which he testified to finding no strychnia. But it is now known that Dr. Taylor is in error in stating that strychnia passes quickly out of the tissues. Messrs. Rogers and Girdwood (*Lancet*, for Oct., 1859) have "demonstrated a singular persistence in their original shape long surviving the processes of decomposition and putrefaction in the tissues with which they are immaturely combined," of strychnia and other poisons.

Again, Taylor says, "Curara, which has, when swallowed, but a slight action, would, however, give the chemical relations of strychnia in the stomach," *op. cit.* p. 680. If this be so, there is then no distinctive test for strychnia. Not being a chemist, we were unable to go into this subject fully, but the facts we have stated. The error into which Mr. Taylor fell in quoting the testimony in the case of Green, lead us to believe that where human life and reputation are at stake, better chemical authority than Dr. Taylor should be demanded.

Are the analyses generally furnished in such cases more reliable? We fear not. The public authorities in this city generally go on the plan of economy, and the cheapest doctor has generally been employed to make post mortem examinations for the coroner, and no chemical examination is sought except in cases like that of Greene, where public opinion demands it. In case of need, we have the satisfaction of knowing that we have a chemist who is capable, laborious and conscientious, whose skill is unquestioned, but the public authorities would never think of giving him *carte blanche* in a difficult case. We see it stated that in the case of Stephens, Prof. Doremus *received* $3,000 for the analysis, and $600 for a new apparatus.

We deem it a duty, as it is a pleasure, to say that the analysis of Dr. Doremus in that case is more perfect and satisfactory than any other of which we have knowledge, either in Europe or America, and it must be no small relief to a court and jury to be able to have such evidence on which to base their action.

Lest this reference to the public authorities here should be misunderstood, we deem it proper to add that it has no reference to former or present public prosecutors, who are capable and indefatigable.

But the case of Jumpertz, in which the theory of the prosecution was, that death was caused by poison, and in which the public authorities had the whole of the body in their hands (except the viscera), and in which, so far as is proven, no chemical examination was demanded, exhibits an ignorance, or a negligence somewhere, which shall not be passed over in silence. If, as is contended, Sophie Werner was poisoned by Henry Jumpertz, with arsenic, strychnia, or any ordinary poison, that poison still exists in the decomposed tissues of the body, it can be brought out, and made visible to court and jury.

Will it be done?

---

PROF. BRAINARD:

*Dear Sir*,—In compliance with your request that I would examine the remarks of Mr. Alfred Taylor, in his last edition of his work on Poisons, in reference to the case of Greene the banker, indicted and convicted in this city in 1854, for the murder of his wife by poison, I send you the following:

The extract referred to will be found on page 696 of Taylor on Poisons, American edition of 1859, and is as follows:

"At Chicago Circuit Court, December, 1854, and January, 1855, G. W. Green, a banker, was charged with the murder of his wife by strychnia. He had been married for many years, and the general evidence showed that he had latterly maltreated the deceased. It seems that she died suddenly while in apparently good health, and no person witnessed her death. The prisoner assigned the cause to cholera, and made some contradictory statements. An inspection of the body by Drs. Myers and Freer, revealed no cause of sudden death; there was no disease. There was lividity about the throat and eyelids, the tongue was protruded, and the limbs were in a rigid state. The stomach, intestines and brain were healthy; the lungs and heart were also healthy; but the heart was empty, as well as the great vessels near it. Dr. Blaney, a chemist, deposed to finding strychnia in the contents of the stomach of deceased. He employed Slas's process, and estimated that the whole quantity which he found amounted to the twentieth part of a grain. He used the color tests, and, having produced some of the crystalline matter in court, he applied the tests for the satisfaction of the jury; one gave the result indifferently and the other not at all. The crystals had a bitter taste. It proved that in the drawer of a bureau, of which the prisoner had the key, strychnia was found; and it is stated, that in the same bureau quinine was kept, which deceased was in the habit of taking for ague, from which she suffered. It was also alleged that deceased herself had used strychnia for poisoning animals. The defence, therefore, turned upon the possibility of deceased having taking the poison (if taken at all) by some mistake, for quinine. Nevertheless the prisoner was convicted. A new trial was moved for and granted.

There may have been other facts of a moral kind, which led to the verdict in this case; but, viewing them as they have been here reported to me, there was a failure of the medical evidence to show that death was caused by strychnia. Nothing was known concerning symptoms; the appearances were of a negative kind, and the chemical analysis, admitting it to its fullest extent, merely proved the evidence of a medical dose of strychnia. In fact, but for this chemical evidence, there would have been no case against the prisoner, and this evidence, in a scientific point of view, was not satisfactory."

It will be observed that Mr. Taylor here makes a wholesale charge, that the conviction was made on insufficient evidence, and also that "this evidence, in a scientific point of view, was not satisfactory." It is the latter charge mainly that we wish to notice.

Any one familiar with the trial of Greene will notice in this statement several important errors, and equally important omissions; and hence it conveys impressions unjust to all the parties concerned in the trial. It is to be deplored than an author of such reputation, and claiming to be an authority on medical legal subjects, should permit himself to pass judgment upon a respectable court and jury, without taking the trouble to inform himself correctly of the facts of the case, especially when it was in his power to do so with but little trouble. He has obviously had his information from some second-hand statement, or from some incorrect, condensed and unauthorized publication of the evidence—such a course cannot but throw doubt on the credibility of his statement of other cases, and depreciate the value of his work as authority. He that attempts to criticise should see that he is not himself subject to criticism.

The first omission of importance is in the statement of appearances after death, viz., that the stomach was filled with food of a recently injested meal, which negatived the theory of the prisoner, that death was the result of cholera, and also the hypothesis of an irritant poison.

In the statement of the chemical evidence, my written report, which was admitted as evidence, and which gives more fully than the oral testimony the various tests, *is not noticed.* Had the author examined this, he would have found that every color test acted distinctly, as made separately and side by side with a known specimen of strychnia. Also that the crystalline forms of the strychnia obtained from the stomach, as examined with the microscope, was the same as of true strychnia. That the chemical habitudes with solvents, of its salts with acids, its bitterness, etc., were all corroborative of the color tests. That these tests were frequently repeated by myself, and in the presence of other parties, and *invariably acted characteristically.*

In regard to the statement of the tests applied in court, there is an important error. He says, "having produced some of the crystalline matter in court, he applied the tests for the satisfaction of the jury. *One gave the result indifferently, and the other not at all.*" This is false in fact. The tests did act, and that distinctly. It is true that one of the tests when applied to a *very minute* fragment of a crystal, though giving the proper color, was so evanescent that time was not allowed to pass it round the jury; but in that case, the *author fails to state* that, on repeating the trial with a larger quantity, *it was strikingly distinctive.* This is distinctly stated in the published evidence. Why does the author not give the whole of the facts? Why does he repress the fact of the repetition of the test when he states the failure in the first trial? Obviously he was not in possession of a true record of the evidence, or his perusal of it was less studied than it should have been, to do justice to the court or the witness.

Certain important omissions occur also in the narrative of the moral evidence, which I have not space to notice, but which acted strongly with the jury.

He states "a new trial was moved and granted." This is not true; the new trial was *refused.* The case was carried up to the Supreme Court, and before decision, *the prisoner hung himself in his cell,* not having been sentenced.

He states that the chemical evidence "merely proved the existence of a medicinal dose of strychnia"—having previously stated that I had estimated the quantity found amounted to the twentieth of a grain. He does not state that my impression was, that I had a larger quantity, but, upon being urged in cross examination to swear to having found a specific quantity, I would not state explicitly that I had more than that amount. The poison was obtained in separate parcels, and I feared to collect them together to weigh them, lest there should be a loss of a part of it. This appeared in evidence. After all, the statement that there was only a medicinal dose discovered, when all circumstances was considered, is but special pleading. It was in evidence, that the contents of the stomach only were operated with, none of the blood, or the tissues remote from the stomach, were included in the analysis. Hence, on the assumption that death resulted from the poison, that portion which produced death, having been removed by absorption, would not be found in the stomach.

Again, the quantity of the contents was over half a pint, and a heterogeneous mixture. Stas's process, as the author well knows, is long and complicated, requiring numerous solutions, filtrations and washing of the residues. Hence, it is scarcely to be expected that the whole quantity of the strychnia could have been eliminated in a sufficient state of purity to give the crystalline form distinctly, and all the color tests. Would Mr. Taylor expect to obtain the *whole* of a medicinal dose of strychnia in the contents of the *full* stomach of an individual who had died from any cause immediately after its administration? Is it reasonable to presume that the medicinal dose was taken by Mrs. Greene immediately before death, or, if so, that I obtained the whole of it? Is it true, then, under all the circumstances, that the chemical evidence "*merely* proved the existence of a medicinal dose of strychnia."

Sufficient, I hope, has been said, to show that, to say the least, Mr. Taylor has been exceedingly remiss in his duties as an author and critic, in his review of the case of Greene.

Yours truly, J. V. Z. BLANEY.

